# Right ventricular function as a predictor of short-term mortality in patients with sepsis and septic shock: an observational study

**DOI:** 10.1186/s43044-022-00316-3

**Published:** 2022-10-20

**Authors:** Ahmed Bendary, Hany Said, Metwally Elemary, Mohamed Mahrous

**Affiliations:** 1 Cardiology Department, Faculty of Medicine, Benha University, Benha, Egypt; 2 Cardiology Department, Ahmed Maher Hospital, Cairo, Egypt

**Keywords:** Right ventricular dysfunction, Sepsis, Mortality

## Abstract

**Background:**

In recent years, attention has shifted to the role of right ventricular (RV) dysfunction in prediction of clinical outcome among patients with septic shock. However, very few studies have correlated RV dysfunction with survival early in the course of sepsis. In the period from September 2021 to July 2022, we included a total number of 248 patients within 24 h of their presentation with sepsis. All patients were subjected to a comprehensive echocardiographic study to evaluate different parameters of RV function and LV systolic and diastolic functions. We aimed primarily to study the predictive value of RV dysfunction on 30-day all-cause mortality rates and ventilator-free days.

**Results:**

Almost half of study population (48.4%) showed evidence of RV dysfunction (in isolation or with LV dysfunction), with 25.4% showing evidence of *isolated* RV dysfunction. Patients with RV dysfunction had a significantly higher APACHE 2 (*P* < 0.001) score and 30-day all-cause mortality rates (*P* = 0.003) compared to those without RV dysfunction. A significant association was reported between 30-d mortality and dysfunction status (*P* = 0.025). Those with no dysfunction had lower mortality (14.1%) than in those with RV dysfunction only (33.3%), LV dysfunction only (20%), and RV + LV dysfunction (31.6%). No significant difference was observed in ventilator free days according to dysfunction status (*P* = 0.081). A multivariate logistic regression analysis showed that RV dysfunction was among the significant independent predictors for 30-day mortality (OR 2.01, 95% CI 1.07–3.81, *P* = 0.031), controlling for the effect of age and gender.

**Conclusions:**

In a cohort of ICU patients with early sepsis, RV dysfunction is found to be common and predictive of 30-day mortality irrespective to the LV function.

## Background

Sepsis or septic shock is a clinical syndrome characterized by augmented and dysregulated immune-inflammatory response to infection, that could culminate finally into what’s called “multi-organ dysfunction” [1]. One of the complications of sepsis is a form of cardiomyopathy called “septic cardiomyopathy” [2]. Classically, both left ventricular (LV) systolic and diastolic functions were the main focus of studies evaluating the impact of septic cardiomyopathy on clinical outcome in patients with sepsis. However, in recent years, attention has shifted to the role of RV dysfunction in prediction of clinical outcome among those patients [3].

The ventricular interdependence is an interesting phenomenon in which the RV can keep pace with the LV for proper overall heart pump function. However, in sepsis, the RV affection in terms of decreased preload and increased afterload adversely affects the RV ability to be in concordance with the LV [4].

Earlier and recent studies evaluating the RV function in patients with sepsis had several limitations including but not limited to the fact that they had no correlation with survival [5, 6], and they evaluated the RV later in the course of sepsis. Moreover, the RV is relatively more challenging for imaging compared to the LV [7], leading to ambiguity in a consensus on which prognostic parameters to use for assessment of RV in septic shock. Taking into consideration the above-mentioned facts and challenges, we thought that it would be of particular interest if we could provide some data on the predictive value of RV function as assessed by echocardiography on clinical outcomes (including 30-day mortality) in patients early during sepsis.

## Methods

### Study design and overview

In this prospective observational study, we aimed primarily to study the predictive value of RV functional parameters as assessed by echocardiography on 30-day all-cause mortality (*primary clinical outcome*) and ventilator free days (*secondary clinical outcome*). We included patients who met the following inclusion criteria:

- Age 20–70 years.
- Smokers and nonsmokers.
- Had a clinically suspected infection.
- Had two or more systemic inflammatory response syndrome criteria.
- Had either septic shock (systolic blood pressure < 90 mm Hg despite an intravenous fluid challenge of > 20 mL/kg or infusion of any dose of vasopressor medications) or severe sepsis (defined in this study as serum lactate > 4 mmol/L) [2].

To ensure inclusion of patients earlier in the course of sepsis, we excluded patients presenting 24 h after onset of sepsis, patients with cardiogenic shock, patients known to have RV failure, and patients with valvular heart disease or congenital heart disease. Patients (or their assigned authoritative family members) who refused to sign an informed consent were also excluded as well.

### Sample size calculation

Sample size was calculated by using Power and sample size software version 3 based on a previous study done by Michael et al. [8], who reported 28 days mortality of 31% in those with RV dysfunction and 16% in those without RV dysfunction. Total sample size was calculated as, 248 cases (120 with RV dysfunction and 128 without). Alpha and power were adjusted at 0.05 and 0.8, respectively.

### Study variables

A. Clinical and laboratory data:
  Mean arterial pressure (mmHg), temperature (celsius), PH, heart rate (bpm), respiratory rate (cycles/min), sodium (meq/L), potassium (meq/L), creatinine (mg/dl), acute renal failure (Yes/No), hematocrite (%), WBC count, Glasgow Coma Scale, FiO2 (> 50%/<50%), APACHE 2 score [9], amount of fluid received in the 6 h before echo (ml), receipt of vasopressors during echo (Yes/No), norepinephrine equivalent dose (in those receiving vasopressors) (ug/kg/min), ventilated during echo (Yes/No), PaO2/FiO2 ratio.
B. Echocardiographic data:
  i. Data for RV:
    TAPSE (cm), RV end-systolic area (cm2), RV end-diastolic area (cm [2]), RV fractional area change (%), RV dysfunction (Yes/No).
  ii. Data for LV systolic function:
    LVEF (%), LV global longitudinal strain (GLS) (%), LV systolic dys-function (Yes/No).
  iii. Data for LV diastolic function:
    LV E/E', LV diastolic dysfunction (Yes/No)

### Methodology of echocardiographic measurements

Transthoracic Echocardiography (TTE) was performed using a GE VIVD S5 machine for clinical indications. Patients were excluded if their TTE occurred more than 24 h after onset of sepsis. All TTEs were performed by a registered diagnostic cardiac sonographer. Studies were interpreted and formatted by an advanced cardiac sonographer.

We measured common echocardiographic parameters, including LV ejection fraction (EF), myocardial performance index, RV fractional area change (FAC), and tricuspid annulus systolic plane excursion (TAPSE) from M-mode, among others. We adhered to imaging standards as recommended by the American Society of Echocardiography [10]. We used Image Arena (TomTec) to perform\speckle tracking for ventricular strain. We selected standard apical four-chamber views for strain analysis. We selected the best available single cardiac cycle regarding image quality and measured longitudinal strain of the endocardium. We rejected images because of poor image quality if we could not track two or more adjacent segments in the apical four-chamber view. For RV strain, we measured the free wall, excluding the septum. All echocardiographic interpretations were blinded to all clinical data at time of image analysis. Clinicians did not follow any specific hemodynamic management protocol based on echocardiographic findings.

Before analyzing any data, we defined RV dysfunction as having either an RV FAC of < 35% or a TAPSE of < 1.6 cm. We selected these parameters based on their ease of measurement and their ubiquity in clinical practice, and we chose thresholds based on published guidelines.

- RV dysfunction was defined as having either an RV FAC of < 35% or a TAPSE of < 1.6 cm.
- LV systolic dysfunction was defined as an EF < 45% or global longitudinal strain of -19% or higher (higher means -18 is higher than -19).
- LV diastolic dysfunction was defined as patients with E/E' > 13.

### Statistical methods

Data management and statistical analysis were done using SPSS version 28 (IBM, Armonk, New York, United States). Quantitative data were assessed for normality using Kolmogorov–Smirnov test and direct data visualization methods. According to normality, quantitative data were summarized as means and standard deviations or medians and ranges. Categorical data were summarized as numbers and percentages. Quantitative data were compared according to 30-d mortality or RV dysfunction using independent t test or Mann–Whitney U test for normally and non-normally distributed quantitative variables, respectively. Ventilator-free days were compared according to dysfunction status using the Kruskal Wallis test. All categorical data were compared using the Chi-square test. All statistical tests were two-sided. P values less than 0.05 were considered significant.

## Results

### Demographics, clinical, and echocardiographic characteristics

In the period from September 2021 to July 2022, we included a total number of 248 patients meeting the inclusion criteria. Thirty-day all-cause mortality was reported in 60 patients (24.2%). After they have been categorized according to their vital status at 30-days, the non-survivors experienced significantly higher age (56 ± 10 years vs. 47 ± 13 years, *P* < 0.001), creatinine (median = 2.9 mg/dl vs. 1.05 mg/dl, *P* < 0.001), acute renal failure (93.3% vs. 28.2%, *P* < 0.001), WBC count (23.2 ± 5.5 vs. 18.8 ± 3, *P* < 0.001), FIO2 > 50% (65% vs. 31.4%, *P* < 0.001), APACHE 2 (median = 31 vs. 6, *P* < 0.001), receipt of vasopressors during echo (93.3% vs. 17%, *P* < 0.001), norepinephrine equivalent dose (median = 1.2 vs. 0.7 ug/kg/min, *P* < 0.001), ventilation during echo (48.3% vs. 2.1%, *P* < 0.001). In contrast, they had significantly lower mean arterial pressure (52 ± 10 vs. 70 ± 11 mmHg, *P* < 0.001), PH (7.2 ± 0.07 vs. 7.37 ± 0.08 mmHg, *P* < 0.001), sodium (131 ± 5 vs. 137 ± 3 meq/L, *P* < 0.001), hematocrit (29 ± 4 vs. 35 ± 4%, *P* < 0.001), Glasgow Coma Scale (median = 9 vs. 15, *P* < 0.001), PaO2/FiO2 ratio (289 ± 46 vs. 335 ± 27, *P* < 0.001) (Table 1).

**Table 1:** Demographics, clinical, and echocardiographic characteristics stratified by vital status at 30-days

	30-d mortality		*P* value
	Yes (n = 60)	No (n = 188)	
*Baseline demographics & clinical criteria*			
Age (years)	56 ± 10	47 ± 13	< 0.001*
Gender			
Males	29 (48.3)	99 (52.7)	0.559
Females	31 (51.7)	89 (47.3)	
Mean arterial pressure (mmHg)	52 ± 10	70 ± 11	< 0.001*
Temperature (°)	37.9 ± 0.5	38 ± 0.3	0.073
PH	7.2 ± 0.07	7.37 ± 0.08	< 0.001*
Heart rate (bpm)	103 ± 7	102 ± 5	0.469
Respiratory rate (cycles/min)	19 ± 3	20 ± 3	0.686
Sodium (meq/L)	131 ± 5	137 ± 3	< 0.001*
Potassium (meq/L)	3.9 ± 1	4 ± 0.6	0.336
Creatinine (mg/dl)	2.9 (0.8–5.2)	1.05 (0.6–5.5)	< 0.001*
Acute renal failure	56 (93.3)	53 (28.2)	< 0.001*
Hematocrit (%)	29 ± 4	35 ± 4	< 0.001*
WBC count	23.2 ± 5.5	18.8 ± 3	< 0.001*
Glasgow Coma Scale	9 (3–15)	15 (5–15)	< 0.001*
FiO2			
> 50%	39 (65)	59 (31.4)	< 0.001*
< 50%	21 (35)	129 (68.6)	
APACHE 2 score	31 (12–41)	6 (1–25)	< 0.001*
Fluid received 6 h before echo (ml)	1500 (500–3500)	1500 (500–3000)	0.438
Receipt of vasopressors during echo	56 (93.3)	32 (17)	< 0.001*
Norepinephrine equivalent dose (ug/kg/min)	1.2 (0.5–2.1)	0.7 (0.2–1.5)	< 0.001*
Ventilated during echo	29 (48.3)	4 (2.1)	< 0.001*
PaO2/FiO2 ratio	289 ± 46	335 ± 27	< 0.001*
Ventilator-free days (days)	0 (0 – 8)	30 (24–30)	< 0.001*
*Baseline echocardiographic criteria*			
TAPSE (cm)	17 ± 3.7	19.5 ± 4.1	< 0.001*
RV end-systolic area (cm^2^)	15.83 ± 4.97	14.13 ± 3.23	0.015*
RV end-diastolic area (cm^2^)	21.87 ± 4.53	21.24 ± 3.94	0.332
RV fractional area change (%)	30.91 ± 8.38	34.96 ± 8.51	0.001*
LVEF (%)	58.1 ± 13.6	64.8 ± 7.8	< 0.001*
LV global longitudinal strain (GLS) (%)	− 18.9 ± 4.5	− 19.5 ± 3.3	0.365
LV E/E'	9.39 ± 4.19	7.82 ± 3.03	0.009*

LVEF, left ventricular ejection fraction; WBC, white blood cells; TAPSE, Transannular plane systolic excursion; LV, left ventricular; RV, right ventricular

*Significant; Data were presented as mean ± SD, median (min–max), or number (percentage)

Regarding baseline echocardiographic criteria, the non-survivors had significantly higher RV end-systolic area (15.83 ± 4.97 vs. 14.13 ± 3.23 (cm2, *P* = 0.015) and LV E/E' (9.39 ± 4.19 vs. 7.82 ± 3.03, *P* = 0.009). In contrast, they had significantly lower TAPSE (17 ± 3.7 vs. 19.5 ± 4.1 cm, *P* < 0.001), RV fractional area change (30.91 ± 8.38 vs. 34.96 ± 8.51%, *P* = 0.001), LVEF (58.1 ± 13.6 vs. 64.8 ± 7.8%, *P* < 0.001) (Table 1).

No significant differences were reported regarding gender (*P* = 0.559), temperature (*P* = 0.073), heart rate (*P* = 0.469), respiratory rate (*P* = 0.686), potassium (*P* = 0.336), fluid received 6 h before echo (*P* = 0.438), RV end diastolic area (*P* = 0.332), LV global longitudinal strain (*P* = 0.365) (Table 1).

### Ventricular dysfunction subtypes

**Fig. 1:**
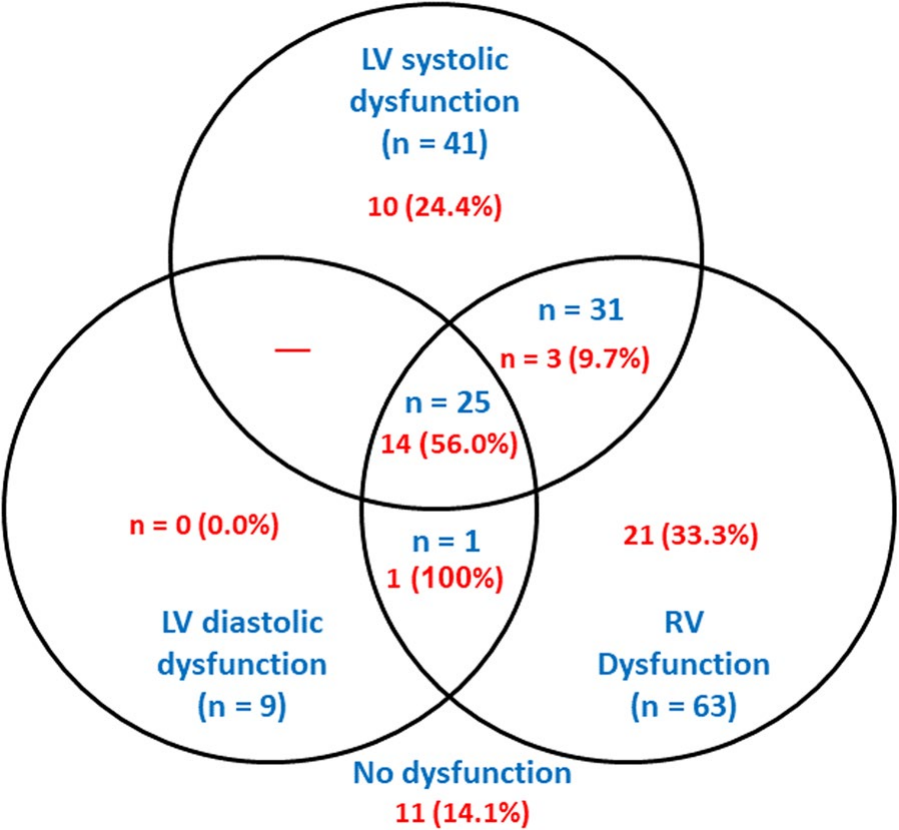
Ventricular dysfunction subtypes among study population (with 30-day mortality expressed in red-colored font)

About one-quarter of the patients had RV dysfunction (25.4%). LV systolic and diastolic dysfunction represented 16.5% and 3.6%, respectively. Only 12.5% had LV systolic dysfunction and RV dysfunction, and one patient had RV dysfunction and LV diastolic dysfunction. Twenty-five patients (10.1%) had LV systolic and diastolic dysfunction and RV dysfunction. About one-third of the patients had no dysfunction (31.5%) (Fig. 1).

### Clinical characteristics and outcomes according to RV dysfunction

Patients were classified according to RV dysfunction. About half of the patients had RV dysfunction (48.4%). Those with RV dysfunction had significantly higher potassium (4.1 ± 0.8 vs. 3.9 ± 0.6 meq/L, *P* = 0.02), creatinine (median = 1.8 vs. 1.1 mg/dl, *P* = 0.004), acute renal failure (50.8% vs. 37.5%, *P* = 0.035), WBC count (20.7 ± 4.8 vs. 19 ± 3.4, *P* = 0.002), FIO2 > 50% (45.8% vs. 33.6%, *P* = 0.049), APACHE 2 score (median = 13 vs. 7, *P* < 0.001), receipt of vasopressors during echo (42.5% vs. 28.9%, *P* = 0.025), and 30-d mortality (32.5% vs. 16.4%, *P* = 0.003). In contrast, those with RV dysfunction demonstrated significantly lower PH (7.31 ± 0.11 vs. 7.35 ± 0.1, *P* = 0.003), sodium (135 ± 5 vs. 137 ± 4 meq/L, *P* < 0.001), and fluid received 6 h before echo (median = 1000 vs. 2000 ml, *P* < 0.001) (Table 2).

**Table 2:** Clinical characteristics and outcome according to RV dysfunction

	RV dysfunction		*P* value
	Yes (n = 120)	No (n = 128)	
Temperature (°)	37.9 ± 0.3	38 ± 0.3	0.085
PH	7.31 ± 0.11	7.35 ± 0.1	0.003*
Heart rate (bpm)	103 ± 6	102 ± 5	0.3
Respiratory rate (cycles/min)	20 ± 3	20 ± 2	0.858
Sodium (meq/L)	135 ± 5	137 ± 4	< 0.001*
Potassium (meq/L)	4.1 ± 0.8	3.9 ± 0.6	0.02*
Creatinine (mg/dl)	1.8 (0.7–5.5)	1.1 (0.6–5.4)	0.004*
Acute renal failure	61 (50.8)	48 (37.5)	0.035*
Hematocrit (%)	33 ± 5	34 ± 4	0.07
WBC count	20.7 ± 4.8	19 ± 3.4	0.002*
Glasgow Coma Scale	15 (3–15)	15 (3–15)	0.542
FiO2			
> 50%	55 (45.8)	43 (33.6)	0.049*
< 50%	65 (54.2)	85 (66.4)	
APACHE 2 score	13 (1–41)	7 (1–34)	< 0.001*
Fluid received 6 h before echo (ml)	1000 (500–3500)	2000 (500–3000)	< 0.001*
Receipt of vasopressors during echo	51 (42.5)	37 (28.9)	0.025*
Norepinephrine equivalent dose (ug/kg/min)	1.1 (0.2–2.1)	0.7 (0.2–2)	0.104
Ventilated during echo	18 (15)	15 (11.7)	0.447
PaO2/FiO2 ratio	319 ± 41	328 ± 34	0.051
30-d mortality	39 (32.5)	21 (16.4)	0.003*
Ventilator-free days (days)	30 (0–30)	30 (0–30)	0.089

WBC, white blood cells; FiO2, fraction of inspired O2

*Significant; Data were presented as mean ± SD, median (min–max), or number (percentage)

No significant differences were observed regarding temperature (*P* = 0.085), heart rate (*P* = 0.3), respiratory rate (*P* = 0.858), hematocrit (*P* = 0.07), Glasgow Coma Scale (*P* = 0.542), norepinephrine equivalent dose (*P* = 0.104), ventilation during echo (*P* = 0.447), PaO2/FiO2 ratio (*P* = 0.051), and ventilator free days (*P* = 0.089) (Table 2).

### Outcome according to dysfunction status

**Fig. 2:**
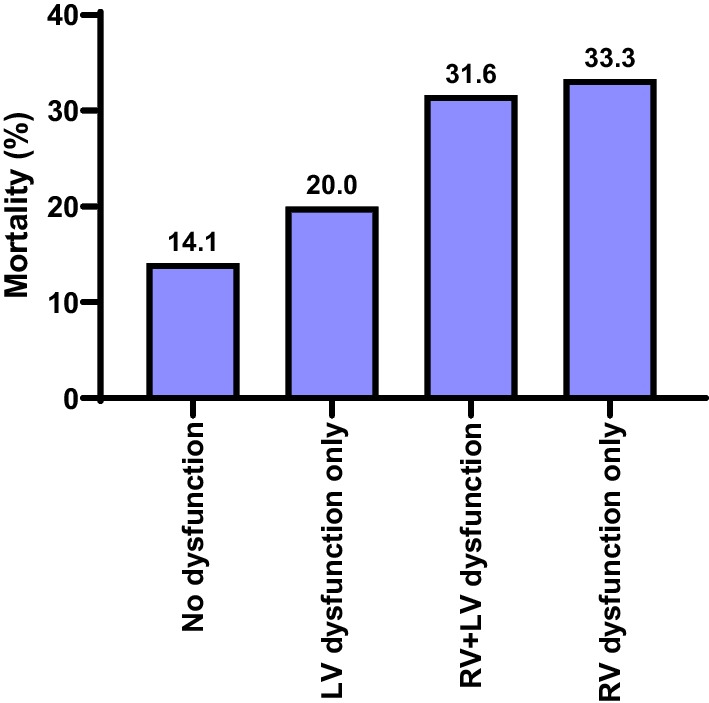
Mortality according to ventricular dysfunction subtypes

A significant association was reported between 30-d mortality and dysfunction status (*P* = 0.025). Those with no dysfunction had lower mortality (14.1%) than in those with RV dysfunction only (33.3%), LV dysfunction only (20%), and RV + LV dysfunction (31.6%). No significant difference was observed in ventilator free days according to dysfunction status (*P* = 0.081) (Table 3, Fig. 2).

**Table 3:** Outcome according to ventricular dysfunction subtypes

	No dysfunction (n = 78)	RV dysfunction only (n = 63)	LV dysfunction only (n = 50)	RV + LV dysfunction (n = 57)	*P* value
30-d mortality	11 (14.1)	21 (33.3)	10 (20.0)	18 (31.6)	0.025*
Ventilator-free days (days)	30 (0–30)	30 (0–30)	30 (0–30)	30 (0–30)	0.081

RV, Right ventricular; LV, Left ventricular

*Significant; Data were presented as number (percentage) or median (min–max)

### Predictors of 30-day mortality

Multivariate logistic regression analysis was done to predict 30-d mortality. It revealed that APACHE 2 (OR 1.38, 95% CI 1.26 – 1.52, *P* < 0.001), RV dysfunction (OR 2.01, 95% CI 1.07–3.81, *P* = 0.031), LV diastolic dysfunction (OR 2.38, 95% CI 1.07 – 5.26, *P* = 0.033), ventilation during echo (OR 97.36, 95% CI 24.49–387.13, *P* < 0.001), and vasopressors need during echo (OR 64.23, 95% CI 20.97–196.73, *P* < 0.001) were significant predictors for 30-d mortality, controlling for the effect of age and gender (Table 4).

**Table 4:** Multivariate logistic regression analysis to predict 30-d mortality

	OR (95% CI)**	*P* value
APACHE 2	1.38 (1.26–1.52)	< 0.001*
RV dysfunction	2.01 (1.07–3.81)	0.031*
LV systolic dysfunction	1.23 (0.65–2.34)	0.521
LV diastolic dysfunction	2.38 (1.07–5.26)	0.033*
Ventilated during echo	97.36 (24.49–387.13)	< 0.001*
Vasopressors need during echo	64.23 (20.97–196.73)	< 0.001*

OR, Odds ratio; 95% CI, 95% confidence interval

*Significant

**Adjusted for age and gender

## Discussion

In the current study, we demonstrated that RV dysfunction (either isolated or in combination with LV systolic or diastolic dysfunction) is not uncommon in patients with sepsis, with RV dysfunction solely associated with significantly higher 30-day all-cause mortality in a multivariate adjusted model.

Our findings are similar to a prior work by Vallabhajosyula et al. [11] who demonstrated that RV dysfunction is common in patients with septic shock. However, the increased mortality in Vallabhajosyula et al. work was observed at 1-year follow-up timepoint (not in the short term). This discrepancy may be explained by the fact that they studied patients later in their course (about 72 h after presentation) and this could have introduced the so-called survivor bias to the analysis for the short-term.

The pathophysiology of the observed RV dysfunction in patients with sepsis (in our cohort and in others) is suspected to be through increased cardiomyocyte cellular dysfunction within the dysregulated humoral and metabolic milieu in sepsis. However, some other postulated mechanisms deserve mention:

1. Since RV function is affected by the afterload imposed by the pulmonary circulation, RV dysfunction may be just an indirect indicator of the severity of the lung disease, which is, in and of itself, the direct actual cause of mortality in those patients.
2. The mortality in our patients may be directly caused by catecholamines administration that have led to RV dysfunction, making RV dysfunction to appear as a cause for increased mortality but in fact it was a bystander. Actually, vasopressor use during echocardiography was shown in our cohort to be significantly predictive of mortality (Table 4).

Therefore, the presence of RV dysfunction in septic shock may be just a consequence of abnormal neurohumoral and metabolic factors such as overuse of vasopressors and fluid administration during the process of sepsis, and not an intrinsic cardiac problem. This is of particular clinical importance for physicians who frequently use the RV functional parameters to adjust medications and fluids for those patients.

The finding that LV systolic dysfunction did not predict 30-day mortality in our population (in contrast to LV diastolic or RV dysfunction) is quite surprising. This is also in contrast to other data that showed an association between LV systolic dysfunction and mortality [12]. We speculate that the thresholds selected in our study to define LV systolic dysfunction (*EF* < *45% or global longitudinal strain of -19% or higher*) may have reflected a group with relatively milder form of LV systolic dysfunction. On the other hand, our used thresholds for RV dysfunction (*having either an RV FAC of* < *35% or a TAPSE of* < *1.6 cm*) may have reflected a considerable degree of RV dysfunction that was sufficient enough to be associated with poor survival.

In contrast to Michael et al. [8] who did not report an association between LV diastolic dysfunction and mortality, we observed that LV diastolic dysfunction is associated with more that doubling in the odds of 30-day mortality [OR 95% CI 2.38 (1.07–5.26), *P* = 0.033]. This could be explained by the higher APACHE 2 score, the lower prevalence of *isolated* LV diastolic dysfunction in Michael et al.’s cohort.

The current study is not without limitations. First, the observational nature of the study hinders any causal claims behind the link of RV dysfunction and survival in patients with sepsis. Second, we did not use RV strain as an assessment tool for RV function, but this could be relatively understood if we know that there is a gap of knowledge about the normative data from larger cohorts for RV strain that could be used as cutoffs to define RV dysfunction in those patients. Third, the vast majority of our patients presented earlier in the course of sepsis than late. We were expecting that ventilator-free days will be sewed to a higher value representing the whole length of hospital stay and follow-up period, as those patients were presenting earlier i.e., relatively healthier. That’s why we assigned it as a secondary outcome. And this was exactly the case when we were analyzing the data to generate the results. We found that the ventilator-free days value was non-normally distributed i.e., skewed to a higher value, and the maximum was the most frequentist (median 30 [range 0–30]), reflecting the relatively healthier patient effect. This prevented us from using it as a continuous variable of meaningful predictive ability or dichotomizing it into 2 categories, as it will yield non-comparable groups and non-valid statistical results. Finally, all our study patients are medical ICU ones, so our findings may not be extrapolatable to other categories of patients.

## Conclusions

In a cohort of ICU patients with early sepsis, RV dysfunction is found to be common and predictive of 30-day mortality irrespective to the LV function. Extreme caution should be exercised before using RV dysfunction improvement as a target for improving outcomes of those patients in general.

## Data Availability

All data are available upon request from the corresponding author(s).

## Abbreviations

FAC: Fractional area change
FiO2: Fractional inspired oxygen
LVEF: Left ventricular ejection fraction
RV: Right ventricle
TAPSE: Transannular plane systolic excursion
WBC: White blood cells
